# Enhancing patient well-being in oncology waiting rooms: a pilot field experiment on the emotional impact of virtual forest therapy

**DOI:** 10.3389/fpsyg.2024.1392397

**Published:** 2024-05-10

**Authors:** Filip Halámek, Miroslav Světlák, Tatiana Malatincová, Jana Halámková, Alena Slezáčková, Zdeňka Barešová, Monika Lekárová

**Affiliations:** ^1^Department of Medical Psychology and Psychosomatics, Faculty of Medicine, Masaryk University, Brno, Czechia; ^2^Department of Comprehensive Cancer Care, Masaryk Memorial Cancer Institute, Brno, Czechia

**Keywords:** psycho-oncology, forest therapy, emotional well-being, stress reduction, virtual waiting room, valence, arousal

## Abstract

**Introduction:**

This study explores the emotional impact of virtual forest therapy delivered through audio-visual recordings shown to patients in the oncology waiting rooms, focusing on whether simulated forest walks can positively influence patients’ emotional states compared to traditional waiting room stimuli.

**Methods:**

The study involved 117 participants from a diverse group of oncology patients in the outpatient clinic waiting room at the Masaryk Memorial Cancer Institute. Using a partially randomized controlled trial design, the study assessed basic emotional dimensions—valence and arousal—as well as specific psychological states such as thought control, sadness, anxiety, and pain. This assessment used the Self-Assessment Manikin and the modified Emotional Thermometer before and after participants watched three video types (forest, sea, news). Baseline stress levels were measured using the Kessler Psychological Distress Scale (K6).

**Results:**

Participants exposed to forest and sea videos reported significant improvements in emotional valence and reduced arousal, suggesting a calming and uplifting effect. No significant changes were observed in the control and news groups. Secondary outcomes related to anxiety, sadness, and pain showed no significant interaction effects, though small but significant main effects of time on these variables were noted.

**Discussion:**

The findings suggest that videos of forest and sea can be a beneficial intervention in the oncology waiting rooms by enhancing patients’ emotional well-being. This pilot study underscores the potential for integrating virtual mental health support elements into healthcare settings to improve patient care experience.

## 1 Introduction

The hospital waiting room is an inevitable component of outpatient care. Despite hospitals’ best efforts, patients spend a lot of time there on their treatment journey. Patients face a spectrum of emotions in this environment, anticipating positive and negative medical updates and challenging physical examinations. This period significantly influences their personal lives and psychological well-being, bringing back memories of past treatments and exposing them to the suffering of others in the shared waiting room space (Catania et al., 2011). Every visit to the oncologist carries inherent traumatic potential, making it a pivotal and emotionally charged event in the treatment trajectory.

Many patients express a desire to leave the hospital as soon as possible without any unnecessary delays. Waiting can often lead to increased frustration, anger, fear, and anxiety, which can, in turn, intensify the perception of physical pain and negative psychological symptoms (Lamba et al., 2020; Fryburg, 2021). A classic example of this phenomenon is “white coat hypertension,” observed in 15 to 30% of patients (Franklin et al., 2013). Stress in the waiting room naturally impacts patient behavior, potentially negatively influencing the patient–physician interaction as well (Fryburg, 2021).

Even though the waiting room might seem like an inconvenient place, it brings us a valuable opportunity. It’s a space where we can, as mental health experts, engage positively with our patients, help them change their waiting room experience, modulate their emotional state before seeing the doctor (Světlák et al., 2023), and perhaps introduce them to potentially helpful practices for mental health support. This does not imply that patients are coerced into any action, but rather that a space is created with incentives and opportunities that encourage movement in that direction. Various means can be used for this purpose.

Television is a commonly used communication channel in waiting rooms (Fryburg, 2021). Because the human eye instinctively and involuntarily gravitates towards movement, the TV screen plays a crucial role in shaping the waiting room ambiance, capturing greater attention than stationary images or wall decorations (Wolfe and Horowitz, 2017). Typically, clinics choose commercially available programs, including a mix of series, talk shows, news, advertisements, and educational spots specifically addressing topics like prevention of respiratory diseases and hand hygiene. Interventions such as watching comedies and listening to relaxation music have been shown to positively affect cancer patients’ emotional well-being in waiting room settings (Genç and Saritas, 2020; Lai and Amaladoss, 2022), as have natural videos and clips depicting kindness, compassion, or other prosocial interactions (Jo et al., 2019; Fryburg, 2021). Conversely, exposure to news content has consistently negatively impacted current and long-term well-being (e.g., Johnston and Davey, 1997; De Hoog and Verboon, 2020).

In healthcare settings, natural stimuli are commonly presented through various mediums, including photographs (Nanda et al., 2012), 3D images, virtual reality (Scates et al., 2020), and audio-visual recordings of natural landscapes (Watts et al., 2016). Viewing natural scenery generally leads to more relaxed body responses than viewing control stimuli (Jo et al., 2019). Studies also collectively suggest that exposure to natural environments can even foster a range of more complex and nuanced positive states, such as connectedness and hope (Tsutsumi et al., 2017) or gratitude (Algoe et al., 2008).

The forest motives seem to be very strong in their effect on well-being (Hejtmánek et al., 2022), which, in some ways, seems analogical to that of forest therapy. Forest therapy, also known as forest bathing or “shinrin-yoku,” is a practice that involves immersing oneself in nature, particularly in a forest or natural environment, to promote physical, mental, and emotional well-being (Rajoo et al., 2020; Rosalind et al., 2023). It originated in Japan and has become popular worldwide as a form of ecotherapy. The key elements of forest therapy include mindful and intentional engagement with nature, such as walking slowly, observing the surroundings, and using the senses to connect with the natural environment. As recent systematic reviews and meta-analyses show, the practice is believed to have various health benefits, including stress reduction, improved mood, enhanced immune function, and a sense of relaxation and rejuvenation (Rajoo et al., 2020; Grassini, 2022; Rosalind et al., 2023). Forest therapy is often seen as a way to counteract the impact of stressors associated with modern urban life by fostering a deep connection with nature (Ulrich et al., 1991). In the context of comprehensive cancer care, research has shown that forest therapy can notably improve sleep quality in patients with gastrointestinal tract cancers, enhancing overall well-being (Kim et al., 2019). Furthermore, integrated medicine therapies that include forest therapy have been shown to enhance spiritual well-being and reduce cancer-related fatigue in cancer patients (Nakau et al., 2013). Additionally, as adjuvant therapy for women with stage III breast cancer, forest therapy has shown promise in enhancing natural cytotoxicity, a critical factor in cancer recovery (Kim et al., 2015). It has also been effective in reducing depressive symptoms and contributing to emotional health in cancer patients (Rosa et al., 2021). Lastly, a study focusing on gynecological cancer patients indicated that forest therapy can reduce heart rate and salivary cortisol levels, further supporting its potential in enhancing general well-being (Ochiai et al., 2015).

If oncological patients cannot go to the forest while waiting for their oncologist, one option is to bring the forest to them through nature videos or imagery (Vincent et al., 2010; Beukeboom et al., 2012).

As mentioned above, the dominant communication channels in this respect are still TV sets in waiting rooms. However, people nowadays tend to engage more with their smartphones and tablets, and, more recently, the potential of virtual reality is also penetrating the field. Although findings of recent years show that Virtual Reality (VR) is a very good way to simulate forest experience because of its high immersion effect (Tashjian et al., 2017; Scates et al., 2020; Hejtmánek et al., 2022; Reese et al., 2022), the advantages of tablets or smartphones still prevail in a waiting room setting. Tablets are generally more widely available, cost-effective, and require less setup than VR systems. Patients in a waiting room may have varying comfort levels with technology, and tablets offer a familiar and user-friendly interface that requires minimal instruction. Additionally, VR headsets may pose challenges such as hygiene concerns, discomfort for some individuals, and the need for periodic maintenance.

Typically, stimulation materials of nature are static, with patients usually simply observing a landscape. Another option, which increases the immersion effect of the first, is the walking person’s perspective.

The walking person’s perspective on a tablet fundamentally transforms a static display into a dynamic journey of discovery and enhancing engagement. It enhances the sense of presence and connection with nature. This interactive element could evoke a more robust emotional response and a more profound feeling of being in the forest than a more passive viewing experience offered by a traditional documentary. The feeling of immersion is further enhanced by the combination of dynamic images and natural forest sounds (Watts et al., 2016).

Hypothetically, the immersive and interactive experience of seeing a forest on a tablet from a walking person’s perspective can be associated with mirror neurons to some extent. The overarching body of research supports the idea that observing others allows us to directly mirror and simulate their experiences, encompassing not only their physical actions but also their physiological states (e.g., Kilner et al., 2006). As the person engages with the virtual environment, mirroring their movements or perspectives, mirror neurons may contribute to a sense of connection and empathy with the depicted scene. The more realistic and interactive the virtual experience, the more likely mirror neurons may induce a sense of presence and a stronger connection with the virtual environment. In other words, one does not need to be in the forest to be able to have an active predictive simulation map in one’s brain with its psycho-neuro-endocrine consequences in our body within a certain scope.

In our pilot field experiment, we investigated the impact of environmental stimuli on patients’ emotional states in oncology waiting rooms. Specifically, we examined whether watching a video simulating a walk through a forest influences the emotional state of patients awaiting ambulance treatment. Additionally, we compared the effects of the forest imagery with the sea view and contrasted these natural scenes with the more conventional television news broadcasts in waiting rooms. Understanding these effects is crucial for hospital management considering the incorporation of virtual mental health support elements into waiting rooms. The concept of a virtual waiting room as a future direction for enhancing patient care are further discussed in the subsequent sections of this article.

### 1.1 Aims of the study

The main objective of the research is to assess whether a video simulating a walk through a forest can influence the emotional state of patients in a waiting room, specifically the basic dimensions of emotional valence and arousal (primary outcomes), and also their overall experience of anxiety, sadness, pain, and control over their thoughts and feelings (secondary outcomes).

To compare the effects of different visual stimuli, the study also aimed to compare the emotional effects of the forest video with those elicited by viewing a calming sea view and to contrast these natural scenes with the more traditional television news broadcasts typically shown in waiting rooms to compare the above emotional effects.

In our study, we also conducted a supplementary data analysis to explore how the level of stress over the past 30 days, as measured by The Kessler Psychological Distress Scale (Kessler et al., 2002), affects patients’ responses to the actual emotional state in the waiting room and to presented videos. This exploration can partially enhance our understanding of how long-term stress levels influence patients’ emotional states before the oncologist’s appointment.

### 1.2 Hypotheses and research questions

#### 1.2.1 Hypotheses regarding primary outcomes

We expected a significant interaction effect with all outcome variables. Specifically, we expected the video journey through the forest and the sea observation will show a greater reduction of situational arousal (H1) and a greater increase in valence (H2) compared to the news, and control group.

#### 1.2.2 Hypotheses regarding secondary outcomes

The video journey through the forest and the observation of the sea are expected to show a greater reduction of actual sadness (H3), anxiety (H4), and pain (H5) and a greater increase in control over the thoughts and feelings (H6) as compared to the news and control group.

## 2 Materials and methods

### 2.1 Participants

A total of 180 participants were enrolled in the study (63 patients refused to participate in the study, reasons are discussed below). The final sample of 117 participants comprised 96 females and 21 males, with no individuals identifying as other genders. The mean age of participants was 60.1 years (SD = 13.1, range 30–81 years). Regarding the highest achieved education level, 5.1% had only completed primary education, 73.5% had completed secondary education, and 21.4% had a college degree. Of those approached, sixty-three individuals declined to participate, and no data were collected from these individuals due to ethical considerations. The study encompassed patients with various oncological diseases, at different stages of their diagnostic and therapeutic journey. Given the study’s adherence to strict anonymity protocols, detailed investigations into the specific nature of these diseases, their diagnostic stages, or the therapeutic measures being undertaken were not conducted.

#### 2.1.1 Recruitment

The data collection process involved directly approaching patients seated in the waiting area outside the attending physician’s office. Each participant was informed about the objectives and design and signed an informed consent. The engagement with patients was conducted by a clinical psychologist (MS) along with a student assistant (FH), working under direct supervision. To ensure consistency in communication, the research team was trained to adopt a uniform approach when interacting with participants, thereby minimizing variations in communication styles. The data were collected from 14 August to 6 September (Rosalind et al., 2023).

#### 2.1.2 Randomization

Each facilitator (MS, FH) presented different video clips to different participants in a pre-determined sequence, which was generated using a random number generator.^1^ In adherence to this sequence, videos were presented to patients randomly. An item on the randomized list was marked as completed whether or not the patient agreed to participate in the study. While the video sequences were selected and presented randomly, the recruitment of patients was non-random and dependent on the discretionary choice of the recruiter at any given time. This methodological aspect and its potential implications are thoroughly examined in our study’s ‘Limitations’ section.

#### 2.1.3 Study groups and measures

Participants were assigned to one of four groups: Group 1—Forest (*n* = 26, 22.2%), Group 2—Sea (*n* = 33, 28.2%), Group 3—News (*n* = 21, 17.9%), and Group 4—Controls (*n* = 37, 31.6%). There were no statistically significant differences between the groups regarding age and gender distribution. Stress levels were assessed using the Kessler Psychological Distress Scale. The analysis revealed no significant difference in baseline stress levels among the groups (*F* [3, 113] = 1.24, *p* = 0.30). Most participants (*n* = 104, 88.9%) reported no or minimal stress (up to 7 points on the scale), with the remainder indicating moderate stress levels.

All dependent variables in our study were skewed towards well-being, indicating that the majority of patients participating did not experience significant discomfort (Table 1). Notably, sadness, anxiety, and pain demonstrated a very strong positive skew—a majority of respondents rated these variables at the level of 0 before watching the videos. Specifically, 65 individuals (55.6%) reported minimal anxiety, and 77 participants (65.8%) reported minimal levels of both depression and pain. This pattern suggests a generally low level of these negative emotional states among our study population at the outset.

**Table 1:** Descriptive statistics.

Variable	Mean (M)	Standard deviation (SD)	Minimum (Min)	Maximum (Max)	Median (MD)	Skewness	Kurtosis
Kessler	3.32	3.21	0	14	2.00	1.30	1.43
Valence Before	3.36	1.15	1	5	3.00	−0.05	−0.51
Valence After	3.90	1.16	1	5	4.00	−0.62	−0.48
Arousal Before	2.18	1.16	1	5	2.00	0.76	−0.18
Arousal After	1.89	1.17	0	5	1.00	1.13	0.50
Control Before	8.12	2.02	2	10	9.00	−1.09	0.38
Control After	8.36	2.11	0	10	9.00	−1.70	2.91
Anxiety Before	1.88	2.75	0	10	0.00	1.31	0.45
Anxiety After	1.13	2.21	0	10	0.00	2.09	3.62
Sadness Before	1.34	2.37	0	9	0.00	1.80	2.17
Sadness After	0.89	1.81	0	9	0.00	2.42	5.76
Pain Before	1.46	2.52	0	9	0.00	1.68	1.65
Pain After	1.23	2.35	0	9	0.00	2.05	3.31

In our research, we encountered a variety of reasons for non-participation among potential subjects (63 patients refused to participate in the study). Many individuals cited time constraints as a primary factor, with many either awaiting imminent appointments or expressing a general sense of being in a hurry. Health-related issues were also cited, including physical discomforts such as headaches and visual impairments that hindered their ability to participate. A notable proportion of our encounters involved individuals showing a general reluctance or disinterest in the research process. This was often expressed through an unwillingness to engage with questionnaires or view videos, and, in some cases, a preference for rest and mental relaxation was stated. Language barriers, nervousness, and concerns about privacy and personal information contributed to the decision not to participate. Importantly, we observed a subset of individuals who declined to participate without verbal communication. These potential subjects communicated their refusal non-verbally, often through gestures or expressions indicating disinterest or unwillingness to discuss the research. This non-verbal refusal mode underscores the diverse range of responses encountered in the field. It highlights the importance of understanding and respecting how individuals may decline participation in research activities.

### 2.2 Study design

**Figure 1:**
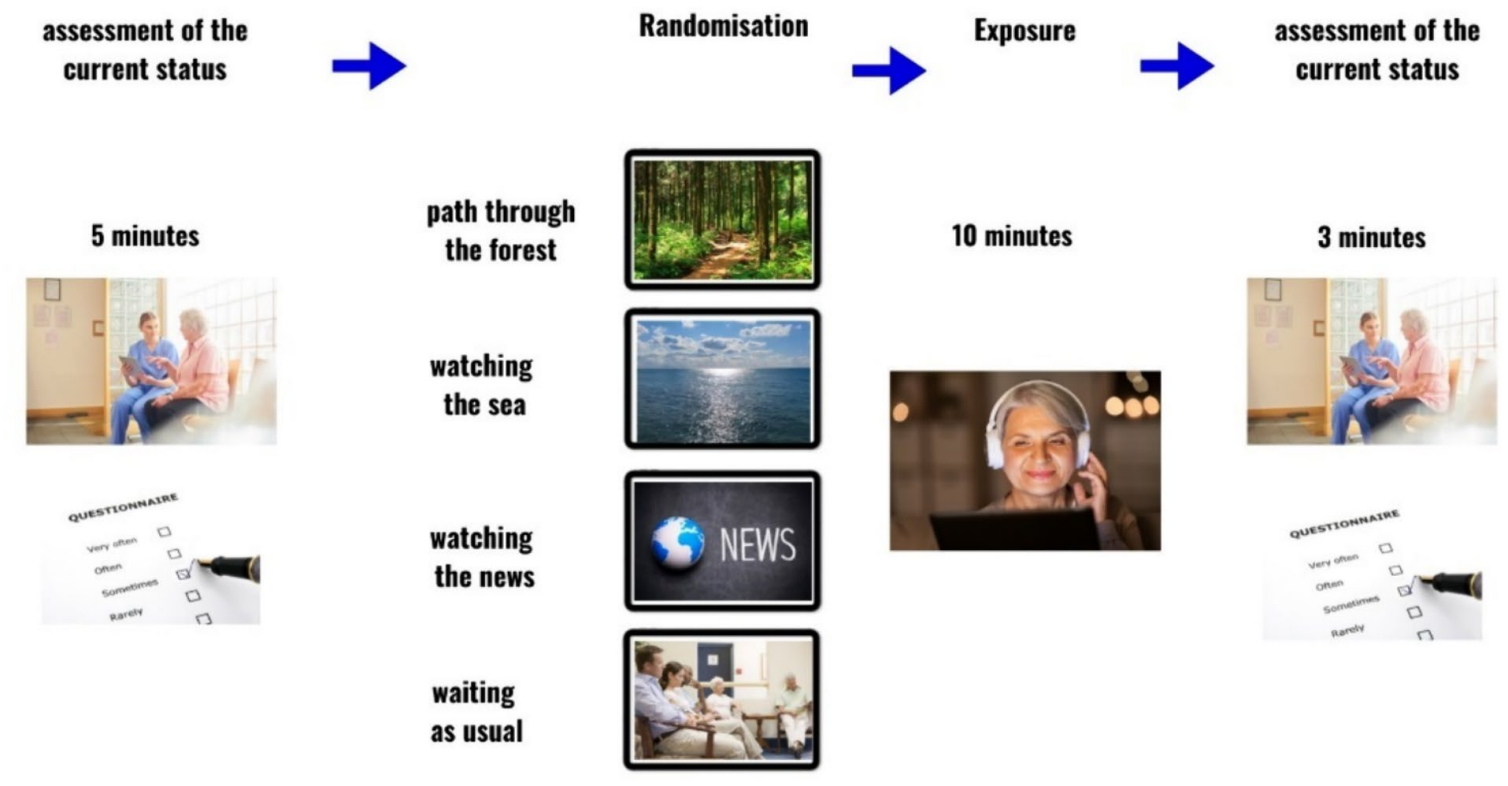
Study design. Prepared using Canva.com.

Considering the randomization process employed, this study may be categorized as a ‘Partially Randomized Controlled Trial’ or as a ‘Randomized Controlled Trial with Non-random Participant Selection.’ The structure and process of the study design are delineated in Figure 1.

#### 2.2.1 Intervention videos

The videos were displayed on a tablet featuring a 10.5-inch Full HD 1920 × 1,200 TFT screen. During the viewing, each patient used wired over-ear headphones. Consistent with the theoretical framework outlined in the introduction of this study, we selected three distinct videos:

##### 2.2.1.1 Walking through the forest

This video involved a walk through an autumn forest, filmed from the perspective of the walker. The footage, set in a highland nature reserve in the Czech Republic, is accompanied by ambient sounds of nature and birds singing.

##### 2.2.1.2 Sea

This video presented a serene scene of the open sea, capturing the gentle movement of waves and the expanse of the sky. The soothing sounds of the sea were included to enhance the viewer’s experience.

##### 2.2.1.3 News

The news segment video featured news coverage relevant to the data collection period. Topics included increasing drought in rural areas, the grape harvest, a fire at a nearby factory, a chemical spill into a river, changes to the parking system in regional towns, and a brief sports update. The format and content of this news segment are typical of those commonly broadcasted in waiting rooms on television.

### 2.3 Outcomes measures

#### 2.3.1 Screening of stress level

To measure stress levels over the past period, the Kessler Psychological Distress Scale K6 was used (Kessler et al., 2002). The K6 consists of six items that inquire about the individual’s emotional experiences over the past 30 days. Each item is typically rated on a 5-point scale, ranging from 0 (“None of the time”) to 4 (“All of the time”). The total score on the K6 is obtained by summing the responses across all six items. Higher total scores indicate a higher level of psychological distress. The K6 is widely used to measure general psychological distress, covering a spectrum of emotional symptoms. It is recognized for its ability to capture symptoms related to depression and anxiety, providing a snapshot of overall mental health. The K6 is commonly employed in research and clinical settings to assess mental health in oncology (Adeyemi et al., 2021; Abdelhadi, 2023; Rosalind et al., 2023). Studies often highlight the diagnostic properties of the K6, indicating its ability to discriminate between individuals with different levels of psychological distress effectively (Kessler et al., 2002).

#### 2.3.2 Current emotional state

**Figure 2:**
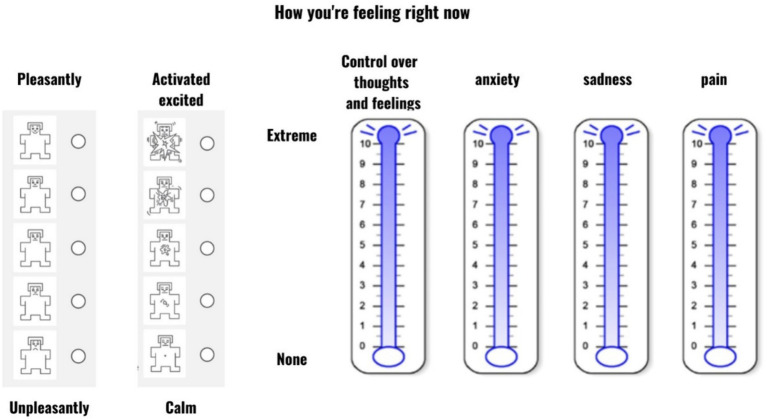
Currant emotional state measurement.

The emotional impact of videos on patients was assessed using the Self-Assessment Manikin (SAM; Bradley and Lang, 1994). The SAM, a method extensively employed in research, is used to evaluate emotional responses and subjective experiences. This tool is particularly effective in quantifying individuals’ affective reactions to diverse stimuli, situations, or interventions. The methodology typically involves participants rating their emotional experiences on two key dimensions using visual scales: pleasure and arousal (Figure 2). These dimensions are fundamental to the emotional space, forming the basis of our conscious emotional experience (Barrett, 2017). The pleasure/valence dimension gauges the extent of positive or negative emotions experienced by an individual. This bipolar scale spans a continuum from highly unpleasant (depicted by an unhappy manikin at the lower end) to extremely pleasant (shown by a happy manikin at the upper end), ranging from 1 (unpleasant) through 3 (neutral) to 5 (pleasant). Arousal is measured similarly, indicating physiological and psychological activation or excitement level (Salgado and Kingo, 2019). Here, participants use the SAM to express their calmness or excitement level in response to the stimulus. The arousal scale, as displayed in the second line of Figure 2, extends from 1 (calm, relaxed manikin) to 5 (extremely excited, aroused manikin). The original dominance subscale was excluded from this study’s design due to its noted unreliability in assessing the impact of emotional stimuli (Bradley and Lang, 1994).

#### 2.3.3 Anxiety, sadness, pain, and control measurement

To assess anxiety, control, sadness, and pain, the modified Emotional Thermometer (Mitchell et al., 2010) was used. The Emotional Thermometer was adapted for the present study. Specifically, we only used the anxiety and sadness subscales from the original instrument and replaced the rest with two additional dimensions of pain and control over thoughts and feelings (Figure 2).

### 2.4 Statistical analysis

To evaluate how the four groups differed on changes observed in the six dependent variables, we conducted a mixed factorial ANOVA in IBM SPSS Statistics, version 29. The expected differences were evaluated using post-hoc comparisons between estimated marginal means with the Bonferroni correction to control for the Type 1 error.

Figure 1 was prepared in the online template editor app for creating presentations Canva. Models were tested separately for each of the six dependent variables. Primarily, we focused on the three basic dimensions of Valence, Arousal, and Control, which exhibited sufficient variance at T0 (before video watching). With Anxiety, Sadness, and Pain, due to the high numbers of participants reporting zero scores at T0, we did not expect significant effects at the time of the analysis. Nevertheless, we still conducted these analyses for the sake of completeness.

## 3 Results

### 3.1 Main results—group comparison

All differences between T0 and T1 ratings (after the video watching) are provided in Table 2. An overview of the main and interaction effects for all dependent variables is provided in Table 3.

**Table 2:** Descriptive statistics of differences between groups.

	All participants		Group 1 forest		Group 2 sea		Group 3 news		Control group	
	M	SD	M	SD	M	SD	M	SD	M	SD
Valence	0.54	1.19	0.69*	1.46	1.06*	1.00	0.33	1.35	0.08	0.80
Arousal	−0.29	0.93	−0.69*	1.16	−0.52*	0.94	0.10	0.83	−0.03	0.60
Control	0.24	1.91	0.46	2.80	0.42	1.52	0.05	1.91	0.03	1.46
Anxiety	−0.75	2.09	−0.81	2.70	−1.18	2.11	−0.52	2.16	−0.46	1.48
Sadness	−0.45	2.00	0.04	2.01	−0.58	1.54	−0.81	2.98	−0.49	1.66
Pain	−0.23	1.11	−0.27	1.78	−0.24	1.03	−0.19	0.51	−0.22	0.82

**Table 3:** The main and interaction effects for all dependent variables.

	Main effect: time of self-report				Main effect: group			Interaction effect: Time × Group		
	*F* (1, 113)	*r*	*p*-value	*η*^2^_partial_	*F* (3, 113)	*p*-value	*η*^2^_partial_	*F* (3, 113)	*p*-value	*η*^2^_partial_
Valence	25.55	0.47	< 0.001	0.18	0.21	0.89	0.01	4.74	0.004	0.11
Arousal	11.55	0.68	< 0.001	0.09	0.79	0.50	0.02	4.91	0.003	0.12
Control	1.73	0.57	0.19	0.02	1.41	0.24	0.04	0.43	0.73	0.01
Anxiety	14.00	0.66	< 0.001	0.11	0.26	0.86	0.01	0.79	0.50	0.02
Sadness	5.84	0.57	0.02	0.05	0.72	0.54	0.02	0.79	0.50	0.02
Pain	4.66	0.90	0.03	0.04	0.47	0.71	0.01	0.02	1.00	0.00

**GRAPH 1:**
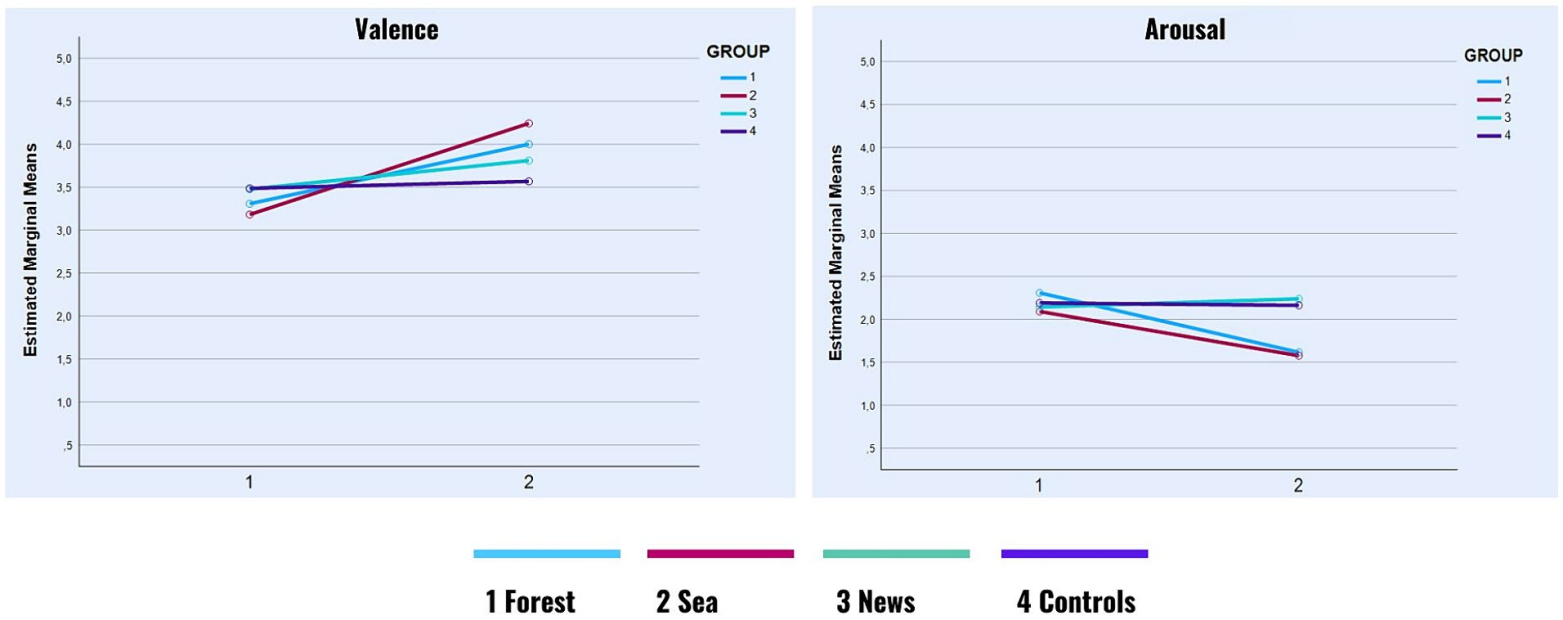
Valence and arousal in time.

Concerning Valence, the expected two-way interaction effect between the group and time of self-report was significant (*F* [3, 113] = 4.74, *p* = 0.004, *η*^2^partial = 0.11). Post-hoc comparisons revealed a significant increase in Valence (i.e., a more positive overall emotional state) between T0 and T1 in the Forest group (ΔM = 0.69, *p* = 0.002, *r* = 0.31, Hedge’s *g* = 0.54) as well as the Sea group (ΔM = 1.06, *p* < 0.001, *r* = 0.59, Hedge’s *g* = 0.96). A slight increase in Valence was also observed in the News group 3 and the Control group 4; however, neither of these differences were significant (Graph 1).

A significant two-way interaction effect was also observed with Arousal (*F* [3, 113] = 4.91, *p* = 0.003, *η*^2^partial = 0.12). Once again, post-hoc comparisons revealed significant differences in the Forest group (ΔM = −0.69, *p* < 0.001, *r* = 0.59, Hedge’s *g* = 0.53) as well as the Sea group (ΔM = −0.52, *p* = 0.001, *r* = 0.63, Hedge’s *g* = 0.49), both of which showed a decrease in Arousal between T0 and T1. In the News group, there was a slight increase in arousal, which, however, was non-significant. In the Control group, the difference between T0 and T1 was negligible (Graph 1).

**GRAPH 2:**
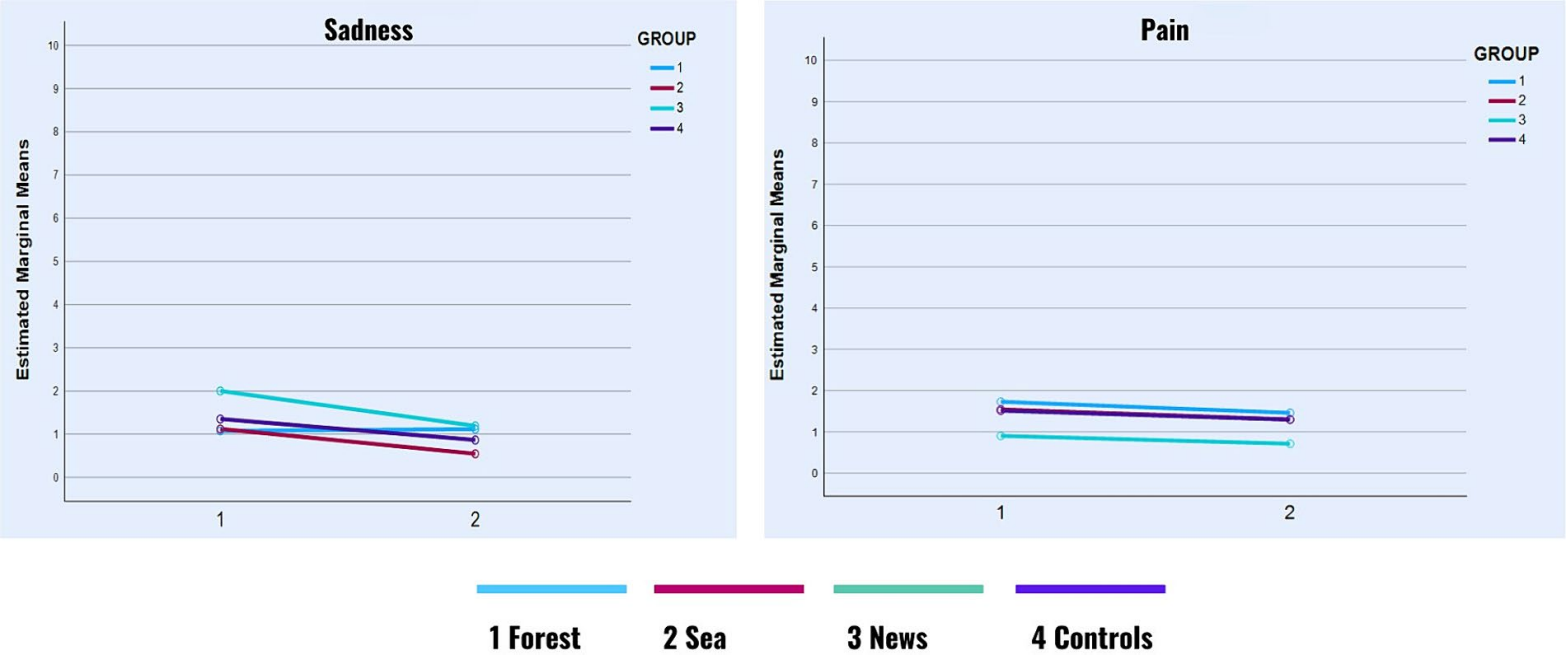
Sadness and pain in time.

**GRAPH 3:**
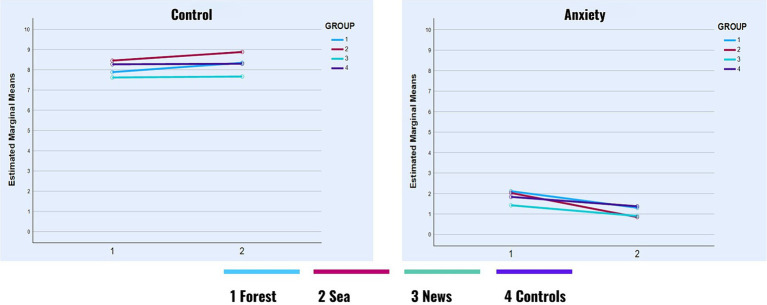
Control and anxiety in time.

None of the hypothesized interaction effects were significant concerning Control, Anxiety, Sadness, and Pain. However, as shown in Table 3, we observed small but significant main effects of time of self-report on Anxiety (Hedge’s *g* = 0.30), Sadness (Hedge’s *g* = 0.21), and Pain (Hedge’s *g* = 0.09), all of which decreased between the first and the second self-report (Graphs 2 and 3) across all groups.

### 3.2 Supplementary analysis

Table 4 presents the results of the supplementary analysis, which explored the correlations between stress levels and emotional reactions to videos at two distinct intervals during the experiment, labeled T0 and T1. The findings revealed a notable pattern. Higher stress levels over the past month were linked to lower valence, indicating that more stressed individuals tended to have less positive emotional responses. In contrast, arousal was positively correlated with stress, meaning that individuals with higher stress levels experienced greater arousal. There was also a negative correlation between stress and perceived control over thoughts and feelings, suggesting that increased stress led to a reduced sense of control. Additionally, stress was positively correlated with anxiety, sadness, and pain, implying that higher stress levels are associated with more intense experiences of these emotions.

**Table 4:** Correlations between all of the dependent variables at T0 and T1 and stress level.

	Valence	Arousal	Control	Anxiety	Sadness	Pain
Kessler (T0)	−0.28^**^	0.35^***^	−0.42^***^	0.47^***^	0.52^***^	0.31^***^
Kessler (T1)	−0.34^***^	0.37^***^	−0.42^***^	0.38^***^	0.33^***^	0.30^***^

*p < 0.05, **p < 0.01, and ***p < 0.001.

At the second time point (T1), this trend continued. The negative correlation between valence and stress became slightly stronger, and while the positive correlations with anxiety and sadness were somewhat weaker, they remained significant. These consistent findings across two-time points underscore the possible predictive power of stress levels in influencing patients’ emotional and psychological responses over time.

A correlation analysis was conducted to understand the interactions among variables, as shown in Table 5. Significant correlations were identified between psychological states at two distinct time points (T0 and T1). Notably, valence was consistently negatively correlated with arousal, anxiety, sadness, and pain, indicating that positive emotional states are inversely related to physiological arousal and negative emotional states. Arousal showed a notable negative correlation with perceived control and a positive correlation with anxiety and sadness, suggesting that increased physiological activation is linked to both a reduced sense of control and heightened negative emotions. Interestingly, control was positively correlated with valence and negatively with anxiety, sadness, and pain, highlighting its pivotal role in emotional regulation. The interplay between anxiety, sadness, and pain was particularly prominent, as these variables were positively correlated, underscoring the interconnected nature of these emotional experiences.

**Table 5:** Correlations between all of the dependent variables at T0 and T1.

	Valence	Arousal	Control	Anxiety	Sadness	Pain
Valence		−0.49^***^	0.32^***^	−0.34^***^	−0.37^***^	−0.25^**^
Arousal	−0.62^***^		−0.26^**^	0.48^***^	0.29^**^	0.23^*^
Control	0.40^***^	−0.41^***^		−0.38^***^	−0.26^**^	−0.18
Anxiety	−0.43^***^	0.48^***^	−0.47^***^		0.51^***^	0.27^**^
Sadness	−0.40^***^	0.48^***^	−0.43^***^	0.60^***^		0.24^**^
Pain	−0.17	0.25^**^	−0.21^*^	0.28^**^	0.26^**^	

*p < 0.05, **p < 0.01, and ***p < 0.001; Correlations at T0 are displayed above the diagonal; correlations at T1 are displayed below the diagonal.

## 4 Discussion

The findings of this study, focusing on the emotional impact of digital landscape video exposure, brought some initial insights into how different types of video content may affect patient well-being in the oncology waiting room. Participants exposed to Forest and Sea videos displayed notable improvements in Valence (a more positive emotional state) and reductions in Arousal, suggesting these natural scenes might have a calming and uplifting effect. In contrast, those who viewed News content or were in the control group showed less pronounced emotional changes. These differences highlight the potential therapeutic benefits of incorporating digital representations of nature into healthcare environments, particularly in waiting rooms where patients might experience anxiety or stress. These insights are crucial for informing future research practices, ensuring they are both effective in data collection and deeply respectful of the participants’ experiences and needs. For a responsible interpretation of presented results, it is necessary to reflect on the fact that our primary data source was derived from self-report measures. Such measures, while practical, often only partially correspond with physiological data collected simultaneously, as evidenced by previous research in this area (e.g., Bailey et al., 2017; Sato et al., 2020). This discrepancy may reflect not only the actual emotional state experienced during the stimulation but also how participants retrospectively interpret or wish to view their emotional responses. As such, the data obtained might provide insights into participants’ expectations or desired feelings post-stimulation rather than their real-time experiences in the waiting room.

### 4.1 Discussion: primary outcomes

#### 4.1.1 Arousal responses to video stimuli: evaluating forest, sea, control, and news (H1)

The study examined the interaction effects on arousal levels across different stimuli, focusing on the Forest, Sea, News, and Control groups. A significant interaction effect was observed, especially when comparing the Forest and Sea groups to the other groups. Notably, both the Forest and Sea groups exhibited reduced arousal over time, measured at various intervals. The decrease in arousal is consistent with the Watts et al. (2016) study results, showing significant improvements in tranquility levels, with a notable increase in tranquility scores using natural sounds and large images of natural landscapes in the waiting room (Watts et al., 2016).

The expected arousal increase within the news exposition was observed but was slight and non-significant. The most likely explanation is that we chose broadcast content that was not particularly exciting or even relevant for the participants.

#### 4.1.2 Valence responses to video stimuli: evaluating forest, sea, control, and news (H2)

The study found a significant interaction effect between group and time of self-report on Valence, indicating a change in overall emotional state. Post-hoc comparisons showed a notable increase in Valence in both the Forest and Sea groups over time. In contrast, the News and Control groups also experienced an increase in Valence, but these changes were not statistically significant. Regarding effect sizes, Hedge’s *g* values were calculated to assess the magnitude of these effects quantitatively. The Forest group demonstrated a Hedge’s *g* value of 0.54 with a medium effect, while the Sea group had a value of 0.96 with a large effect. Thus, we have not confirmed hypothesis H2, and the above-cut effect of passing through the forest from the perspective of a walking person was not demonstrated. On the contrary, our findings show that seeing the sea evokes more pleasant patient feelings than the forest.

While insignificant, the increase in valence over time in news viewing is inconsistent with our expectations. Although specific studies directly examining the impact of news viewing in hospital waiting rooms are limited, there is some evidence about the potential negative impact of less positive or more stressful media, like news broadcasts (Johnston and Davey, 1997; Luo, 2017; De Hoog and Verboon, 2020). One possible explanation for our seemingly counterintuitive findings is the distraction of attention away from the patient’s negative emotional state at the time, which might have had a greater impact than the news content, which usually has a largely negative connotation. For instance, Mehrabi et al. (2015) conducted a systematic review focusing on coping strategies used by breast cancer survivors. The review found that avoidance and distraction were among the strategies used by these patients, indicating their role in coping with the disease (Mehrabi et al., 2015). Luo (2017) conducted a study indicating that positive distractions in waiting rooms, such as interactive games, can influence patient stress levels and perception of wait times. Although nonsignificant, observing an unexpected increase in valence could reflect natural fluctuations over time, the impact of external factors, or even the process of repeated measurement. It can be a result of interaction with the device or the recruiter, too.

#### 4.1.3 Hypotheses regarding secondary outcomes

Valence and arousal were chosen as primary outcomes because of their fundamental role in emotional experience. Contemporary emotion theories reveal that emotions like sadness, anxiety, and pain, as well as how our ability to control emotions and thoughts, are interpretations of basic effects defined by arousal and valence (Barrett, 2017). Thus, emotions like sadness and anxiety and the sense of control over them are considered more complex descriptions, susceptible to individual and cultural variations.

The observed difference between the fundamental aspects of emotion, such as affect (which exhibited significant change), and more intricate emotions, such as sadness or anxiety (which exhibited no change), is partially supported. Watts and his colleagues (2016) present a similar result in their study of the effect of natural videos and sounds in waiting rooms. Their results show that levels of reported tranquility were significantly improved (decrease of arousal in our study), but there were more minor changes in reported reductions in anxiety (Watts et al., 2016).

The observed small but significant main effects of time of self-report on Anxiety (Hedge’s *g* = 0.30), Sadness (Hedge’s *g* = 0.21), and Pain (Hedge’s *g* = 0.09), all of which decreased between the first and the second self-report, can be explained as an effect of adaptation in the situation, distraction, time fluctuation of the emotional state, the positive effect on emotional state induced by researcher’s interest or the process of repeated measurement. Baseline levels of anxiety, sadness, and pain were already so low at the start of the experiment that significant decreases in any of the groups were unlikely. The study’s finding of a small baseline level of negative emotions in T0 among participants is not just due to selection bias. It could also reflect the social desirability of emotion management in our cultural context or highlight that many individuals may not recognize or fully acknowledge their emotional distress. In a medical context, it is much easier to talk about physical symptoms, even though they may be manifestations of anxiety or sadness than to talk about not being able to cope with the situation and needing psychological help.

Given the gender distribution in our sample, where a significant majority of participants are female (96 out of 117), conducting a gender comparison might be challenging and potentially less informative. However, gender differences are to be expected and have an impact. The investigation into sex differences in emotional responses to visual stimuli, across various cultural contexts, revealed notable disparities. Males consistently rated positive pictures as more arousing compared to their female counterparts. On the other hand, females generally reported slightly higher arousal and lower valence ratings for the same stimuli. These findings suggest that males might experience positive images as more emotionally stimulating, while females might perceive these images as less pleasant and more intense (Branco et al., 2023; Rosalind et al., 2023). This difference highlights the importance of considering gender when analyzing emotional responses.

#### 4.1.4 Impact of stress levels on emotional responses to videos

Initial findings indicated comparable stress levels across the four groups, with a predominant inclination towards minimal stress experiences. This baseline condition provided a nuanced understanding of the participant pool, predominantly exhibiting positive well-being indicators. Unfortunately, the last two studies using Kessler’s Psychological Distress Scale do not report average results in their cancer patient cohort (Clover et al., 2009; Sampasa-Kanyinga et al., 2018). However, they report a close significant relationship between the Emotional thermometer and K-10 (Clover et al., 2009), as was documented in our study as well. The supplementary analysis shed light on how pre-existing stress could influence the perception and emotional reaction to stimuli, offering insights into the complex interplay between stress and emotional well-being.

The main clinical impact of these findings is the emphasis on the importance of stress management and intervention strategies within therapeutic settings. By targeting stress reduction, healthcare professionals can indirectly mitigate negative emotional states, enhance patients’ sense of control, and potentially alleviate symptoms of anxiety, depression, and pain.

The circular causal interaction between physical and emotional pain is a clinically relevant fact (Mee et al., 2006; Eisenberger, 2012). The significant interrelations among pain, sadness and anxiety in our study support are consistent with this idea (Table 4). These results only underline the importance of stress screening in cancer patients (Vodermaier et al., 2009).

Our findings also illustrate those confounding variables, such as the prior psychological states of patients and their treatment expectations, could play an important role in influencing the outcomes of this study. Research in this field indicates that the effectiveness of psychotherapeutic interventions for breast cancer patients in a liaison-consultation psychiatry setting is affected by their psychological state and stress perceptions (Grube and Weigand-Tomiuk, 2014). Additionally, a meta-analysis showed that the variability in psycho-oncologic intervention outcomes among cancer patients can be attributed to their treatment expectations and initial levels of distress (Heron-Speirs et al., 2012). Furthermore, studies by Constantino et al. (2018) underscore the significant impact of patients’ outcome expectations on their mental health after treatment, emphasizing the need to consider these expectations in therapeutic strategies. Unfortunately, we do not know anything about our patients’ expectations; however, as will be discussed in the limitations of the study, the intent of the intervention was probably largely obvious to the patients.

Personalized and evidence-informed medicine (Attia and Gifford, 2023) does not seek a one-size-fits-all method, but asks for whom, when, at what stage of the disease is the particular method the best one. Research into mental health promotion in psycho-oncology offers an endless number of effective methods (Breitbart et al., 2023; Rosalind et al., 2023). In this context, we are not faced with the question of what to use, but how to use it and in what narrative and conceptual thinking about mental health support incorporate existing methods and approaches. Our findings reveal that videos of forests and seas positively influence the emotional state of patients in waiting rooms. Building on these results, we suggest offering similar videos in a customizable format, allowing patients to select according to their preferences, with the simple guidance based on research that viewing these videos may be beneficial.

When implementing any mental health support intervention, it’s essential to rigorously assess any possible negative effects on patients. Currently, there are no known adverse effects associated with the use of calming videos of forest and sea. However, these videos are generally considered soothing, it’s necessary to evaluate if they could have unintended negative impacts, such as causing sensory overload or clashing with personal preferences. In the proposed virtual waiting room model outlined in our article, we emphasize the importance of choice in this kind of psychological interventions. Allowing patients to select and dose scientifically validated treatments themselves minimizes potential harm. Likewise, we trust patients in what they look at on their smart devices in waiting rooms, where, in turn, we know about the negative impact of news and certain information on social media (Valkenburg et al., 2021).

## 5 Study limitations

Our current study has several limitations. One source of bias is that the patients in our sample mostly reported low levels of stress and generally did not experience negative emotions such as anxiety, pain, depression, or loss of control at the beginning of the experiment. The reason for this bias lies in the challenges of performing these kinds of experiments in clinical settings. A dual sense of responsibility guided our methodology: respecting and safeguarding the patients’ emotional well-being and confronting and managing our own apprehensions as researchers. Ethically, we were deeply conscious of not wanting to overwhelm patients who might already be experiencing heightened stress or anxiety due to their clinical surroundings. There were instances where it was not feasible or ethically appropriate to approach patients for participation due to their apparently vulnerable or physical state. The possibility of adding to their burden by requesting their participation in a study weighed heavily on our decision-making process. Simultaneously, on a personal level, we grappled with our fears—the fear of rejection and the trepidation of potentially making patients uncomfortable. This fear was not merely a professional hurdle but a reflection of our empathy and respect for the patient’s emotional state. It underscored our commitment to conducting research that is not only scientifically robust but also humanely sensitive.

Another limitation of our study is notably influenced by the absence of subgroup analysis, which restricts our understanding of which specific patient groups benefit most from the intervention. This was primarily due to the design of our study, which did not allow for such detailed analyses. As a result, we are unable to distinctly identify which patients, based on demographic characteristics, cancer type, or treatment stage, find the intervention most useful. This limitation not only constrains the applicability of our results to specific populations but also highlights a critical area for future research. Further studies are needed that are specifically designed to include subgroup analyses to provide deeper insights into the effectiveness of the intervention across diverse patient groups, thereby enhancing its clinical applicability and effectiveness.

A limit that also should be mentioned is the lack of detailed knowledge about what the participants were actually doing while waiting. We categorized this as ‘waiting as usual,’ which involved exposure to environmental cues in the waiting room or engagement in their habitual activities during the waiting periods. This categorization resulted in a very heterogeneous control group, where the only consistent factors were the shared environment of the waiting room and the interactions with the researcher during the initial assessments at T0 and T1. These interactions were identical across all study groups.

### 5.1 Future study design

Another important methodological limitation of our study is that the experiment’s intention was likely obvious to people, which could have significantly biased the results towards a more positive outcome. Although the patients did not know that there were multiple videos to choose from, the pre-and post-exposure measurements encouraged the assumption that the effect of video viewing on the assessed variables was being tested. We tried eliminating this effect by randomizing three different stimulus videos and a non-video control condition. However, one of the best solutions for future research is to ensure that patients are unaware they are participating in an experiment. For example, they could fill out a questionnaire upon arrival in the waiting room with instructions from the nurse that researchers are interested in how patients feel in the waiting room while waiting to see the doctor.

Without being reminded, they would complete the same questionnaire again on their way out (even better, perhaps, when they enter the doctor’s office, i.e., before the actual medical examination). The various content of the videos (forest, sea, news, control) presented on the big screen TVs in the waiting room could be changed on different days or for different patients. At some point after completing the self-report for the second time, the patients might be casually asked additional manipulation check questions by the medical staff (e.g., whether they had noticed the video that was running on the TV screen and how much time they might have spent viewing it while waiting).

## 6 Future directions: innovating patient experience with the concept of a virtual waiting room

In contemporary healthcare settings, the patient experience is pivotal, not only during medical consultations but also during the waiting period. Like many others (e.g., Fryburg, 2021), our results support the creation of the “virtual waiting room” (VWR) concept in the context of the development of eHealth and the availability of smartphones and internet connectivity. The VWR could transcend the traditional idea of a waiting area by offering a range of educational, therapeutic, and relaxation resources accessible through QR codes or web links in the physical waiting area. The VWR could utilize the waiting time effectively by engaging patients in activities that promote physical and mental health. It would include diverse content categories like educational videos, health and wellness podcasts, mindfulness exercises, and interactive learning modules. These resources would be tailored to provide patients with valuable health information, stress reduction techniques, pain management strategies, and tips for effective communication with healthcare professionals. Moreover, the VWR could offer personalized content recommendations, real-time appointment updates, and options for filling out pre-appointment paperwork, thereby streamlining the patient journey. We believe that the intervention and research potential of VWR is enormous, and efforts invested into implementing the idea are not wasted.

The research was originally performed at a cancer institute in the Czech Republic, which benefits from comparatively robust health resource access. However, in developing nations where there might be constraints like insufficient physical infrastructure and limited availability of electronic devices for patients, deploying this intervention could encounter substantial obstacles. Future studies should investigate modified approaches that navigate these limitations, potentially emphasizing interventions that are less dependent on high-tech solutions.

In concluding the future directions section, it is crucial to emphasize that while the adaptability of virtual forest therapy across varying patient demographics is significant, the implementation of innovative concepts like virtual waiting rooms in hospital settings, particularly in developing countries, could be hindered by the need for substantial infrastructure investments and staff training. Future research should focus on low-cost alternatives or smaller-scale integrations, such as in medical offices or specialized clinics, to ensure these interventions are culturally relevant, accessible to all patients, including those with disabilities, and capable of significantly enhancing patient well-being.

## Data availability statement

The raw data supporting the conclusions of this article will be made available by the authors, without undue reservation.

## Ethics statement

The studies involving humans were approved by The Ethics Committee Institutional Review Board of the Masaryk Memorial Cancer Institute approved the research protocol (approval number 2023/1521/MOU; JID MOU 447696; date of approval: June 13, 2023). The studies were conducted in accordance with the local legislation and institutional requirements. Written informed consent for participation in this study was provided by the participants’ legal guardians/next of kin.

## Author contributions

FH: Data curation, Formal analysis, Investigation, Project administration, Writing – review & editing. MS: Conceptualization, Formal analysis, Funding acquisition, Investigation, Methodology, Project administration, Resources, Supervision, Visualization, Writing – original draft, Writing – review & editing. TM: Data curation, Formal analysis, Methodology, Supervision, Visualization, Writing – original draft. JH: Conceptualization, Funding acquisition, Investigation, Methodology, Project administration, Resources, Supervision, Writing – review & editing. AS: Conceptualization, Formal analysis, Funding acquisition, Methodology, Resources, Writing – review & editing. ZB: Conceptualization, Data curation, Formal analysis, Writing – review & editing. ML: Conceptualization, Data curation, Formal analysis, Methodology, Project administration, Writing – review & editing.

## Footnotes

^1^ Random-number-generator.com
